# Investigating farm-level risk factors for *Salmonella* Dublin infection and on-farm transmission in British Columbian dairy farms

**DOI:** 10.3168/jdsc.2025-0884

**Published:** 2025-11-13

**Authors:** Ellen Boyd, Erin Cuthbert, John Dick, David Renaud, Chelsea Himsworth

**Affiliations:** 1 School of Population and Public Health, University of British Columbia, Vancouver, Canada V6T 1Z3; 2 Ministry of Agriculture and Food, Government of British Columbia, Abbotsford, Canada V3G 2M3; 3 Greenbelt Veterinary Services Ltd., Chilliwack, Canada V2P 7Z5; 4 Department of Population Medicine, University of Guelph, Guelph, Canada N1G 2W1

## Abstract

A recent study found that 30% of all dairy farms in British Columbia (BC) were positive for *Salmonella* Dublin, which has prompted a need to determine how to properly manage and mitigate *Salmonella* Dublin within the province. The overarching goal of this study is to use the components of a Danish *Salmonella* Dublin risk scoring tool to perform a cross-sectional, exploratory study to evaluate whether the tool, or components therein, can differentiate between *Salmonella* Dublin bulk tank milk (BTM)-positive versus BTM-negative BC dairy farms. A total of 70 BC dairy producers completed the survey and were classified as positive or negative based of BTM surveillance results. Overall, the components outlined in the modified Danish tool failed to differentiate herds that were positive or negative for *Salmonella* Dublin on BTM, with only 2 risk factors being identified. Farms that reported housing more than one adult dairy cow in the maternity pen were 4 times more likely to be positive for *Salmonella* Dublin on BTM than farms that only kept one adult in the maternity pen at a time. Future research should seek to determine if further modifications to the risk factor survey would be useful, especially the inclusion of questions on factors that target introduction of *Salmonella* Dublin versus on-farm transmission of *Salmonella* Dublin.

## Highlights

• Farms that housed >1 adult dairy cow in the maternity pen were 4 times more likely to be positive for *Salmonella* Dublin on BTM.
• The Danish risk scoring tool failed to differentiate *Salmonella* Dublin-positive versus *Salmonella* Dublin negative herds in BC.
• Alternative outcome measures may be more effective when evaluating risk factors, such as changes in BTM status, individual cow serology, or clinical illness.

## Graphical Abstract

**Figure:**
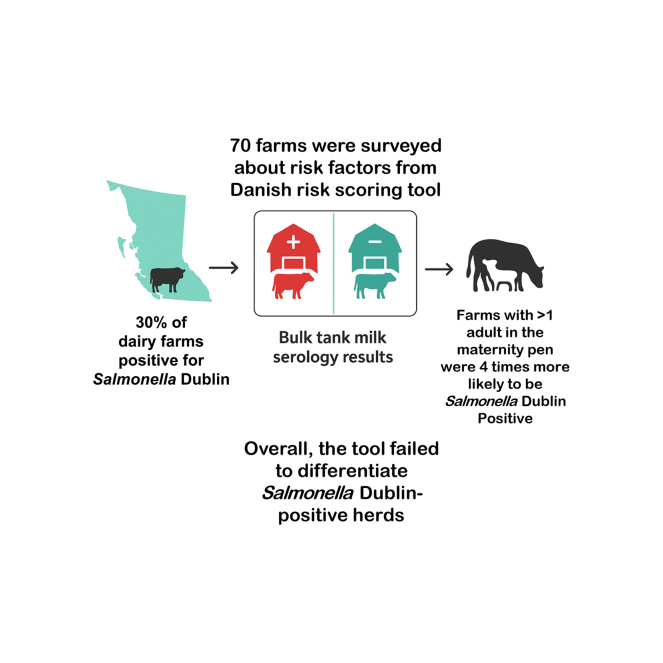
**Summary:** Seventy British Columbia (BC) dairy farms were surveyed about *Salmonella* Dublin risk factors from a Danish risk scoring tool. Farms were classified as possible for negative based on bulk tank milk (BTM) serology status from the province's surveillance program. Farms that often kept >1 adult in the maternity pen at a time were 4 times more likely to be *Salmonella* Dublin positive. However, overall, the tool failed to differentiate *Salmonella* Dublin-positive versus *Salmonella* Dublin-negative herds, which calls into question its utility in the BC dairy context.

*Salmonella* Dublin (serotype D) is a cattle-adapted bacterial pathogen that has profound negative impacts on the dairy industry due to calf mortality, abortion, and reduced milk yield (Nielsen, 2013). In 2002, Denmark initiated a *Salmonella* Dublin surveillance and control program that classified herds as level 1 (most likely unaffected), level 2 (probably infected), and level 3 (bacteria detected; Nielsen and Nielsen, 2006). Approximately 3 years after implementation, 17% of herds were classified as level 2 or level 3, making it difficult to buy or sell cattle because the buyer would receive a temporary restriction on purchasing and be barred from participation in cattle shows (Nielsen and Nielsen, 2006). In response to this alarming trend, a tool was created to identify farm-specific risk factors for *Salmonella* Dublin infection with the goal of helping individual producers identify and modify the specific farm management processes that promote *Salmonella* Dublin introduction and spread (Nielsen and Nielsen, 2006). The tool was initially based on a manual from the United States that addressed paratuberculosis but was modified to focus on management factors relevant to the Danish dairy farming systems that were predicted to be strongly associated with *Salmonella* Dublin in cattle (Rossiter et al., 1999; Nielsen and Nielsen, 2006). This tool, along with the active control measures, helped to reduce the prevalence of *Salmonella* Dublin in Danish dairy herds from 26% to 6% over 8 years (Nielsen and Rattenborg, 2011).

A recent study found that 30% of dairy farms in British Columbia (**BC**) were positive for *Salmonella* Dublin, which was almost twice as high as the prevalence of *Salmonella* Dublin found in other provinces (e.g., Quebec = 7%, Ontario = 5.1%, Alberta = 15.6%; Um et al., 2022; Nobrega et al., 2024; Shaukat et al., 2024; Boyd et al., 2025). This has prompted a need to determine how to properly manage and mitigate *Salmonella* Dublin within the province. To date, the Danish risk scoring tool and other minor modifications are the only known risk scoring tool available for *Salmonella* Dublin, but it is unclear how effective it might be in identifying risk factors within BC. British Columbia and Denmark have many comparable demographics, such as average herd size, with BC having an average herd size of 135 milking cows compared with Denmark's 160 milking cows, breed (predominantly Holsteins), indoor housing, and milk production managed through a fixed quota system (Government of British Columbia, 2022; Danish Agriculture and Food Council, 2024). Given its effectiveness in Denmark, there is merit in determining whether the tool would also be effective in BC. However, despite the similarities, certain practices that are important in Denmark may not be applicable to BC. Because of this, it is important to perform an exploratory analysis using this tool within the BC dairy context.

The overarching goal of this study is to use the components of the Danish *Salmonella* Dublin risk assessment tool to perform a cross-sectional, exploratory study to evaluate whether the tool, or components therein, can differentiate between *Salmonella* Dublin infected versus noninfected BC dairy farms.

A survey was developed using the Danish risk scoring tool so that the components could be evaluated for BC dairy farms (Nielsen and Nielsen, 2006). The survey consisted of a mix of multiple choice and closed- and open-ended questions. Some questions included skip patterns, where certain questions were only shown to producers depending on how they answered previous questions (e.g., if a producer answered that they did not raise their own heifers, they were not asked additional questions on heifer management). All questions pertained to herd demographics, calf management (housing and feeding), biosecurity, and other farm management practices. Questions were refined through consultation with herd health veterinarians, veterinary epidemiologists, and other industry stakeholders. This resulted in the addition of questions that are specifically relevant to the BC dairy producer context (e.g., “do you raise your own replacement heifers?”), and removing questions that were not relevant (e.g., teat cleaning practices within the maternity pen). The survey was pre-tested for clarity and feedback by a BC dairy producer before circulating to all BC dairy producers.

All licensed dairy producers (n = 437) within the province of British Columbia in 2023 were invited via email to participate in an online survey. The survey was administered through Qualtrics (Qualtrics XM, 2023) and was accessible through a direct link from the email. To encourage participation, the first 80 participants were eligible to receive Can$75 upon completion of the survey and correctly answering the attention check question. The attention check question was used to improve reliability of the responses. Only one submission was permitted per licensed dairy farm. Producers were engaged multiple times via email, industry stakeholders (e.g., BC Dairy), and professional contacts (e.g., herd health veterinarians, provincial dairy inspector). After the initial survey responses were received, a second round of recruitment using a modified survey (see below) was conducted. This second survey was deployed to attempt to get more positive farm participants to mirror the true distribution of positive dairy farms in the province (e.g., 30% of the province was positive for *Salmonella* Dublin, and we attempted to have 30% of participants be from positive farms; Boyd et al., 2025). The initial survey administration was active between June 2023 and June 2024, and the second survey was active between September 2024 and December 2024.

A total of 33 potential risk factors that were adapted from the Danish risk scoring tool were included for initial consideration. After the initial round of survey responses (n = 64) were received, some variables were eliminated or combined, and 12 questions were removed because they provided information captured in another question. For example, the questions “How often are tools or equipment used with pre-weaned calves also used with older animals on the farm (e.g., buckets, shovels, medical equipment)?” and “How often are tools or equipment used with weaned calves also used with older animals on the farm (e.g., buckets, shovels, medical equipment)?” were modified to “Do you often (at least once a month) share equipment between different age groups (calves, heifers, adults)?”. This was done because the length of the survey was noted as a deterrent for producers to participate. To improve power, responses of questions (multiple choice, closed-ended) were modified to be a binary variable. For example, a question asking “How many cattle are in the maternity pen at once?” was modified to “Is more than one adult in the maternity pen at any given time? (yes or no).” Those who indicated one adult would be labeled as no, and those who indicated 2 or more would be labeled as yes. A modified version of the survey containing the 21 remaining questions was circulated to *Salmonella* Dublin–positive producers who had not completed the initial survey to get more respondents in order to improve the distribution of positive and negative farms.

Results from a bulk tank milk (**BTM**) surveillance program were used as the outcome variable (*Salmonella* Dublin–positive vs. *Salmonella* Dublin–negative; Boyd et al., 2025). A total of 4 BTM samples were tested from each BC dairy farm between September 2021 and April 2023, with one sample being tested approximately every 3 mo. A sample was considered positive if the percent positivity was ≥35% and a farm was considered positive if any one of the 4 milk samples was positive. A more detailed description of the methodology is available in Boyd et al. (2025). Producers were linked to the BTM surveillance data using information collected during the survey, including their name, farm name, and address.

The goal of the model-building protocol was to identify the most parsimonious set of variables that best explained the outcome of *Salmonella* Dublin positivity, and all variables were given equal consideration going into the model-building procedure (e.g., there were no a priori hypotheses regarding the relative importance of specific predictors). Similarly, there was no a priori hypothesis regarding interactions among variables, and therefore no interaction terms were included for consideration.

To identify risk factors associated with *Salmonella* Dublin status, the distribution of the explanatory variables was examined, and the variables were also examined separately between the positive and negative groups. A *P*-value was generated for each variable using a chi-squared test. Variables with a *P*-value ≤0.2 were considered for inclusion within the multiple logistic regression (**MLR**) model. Backward elimination method based on Akaike information criterion (**AIC**) was used to select the final model. The final MLR model was used to examine relationships between *Salmonella* Dublin–positive farms and each predictor variable.

A total of 64 producers completed the long-form survey, and an additional 6 producers completed the short-form survey (response rate = 16%). Among the 70 participating producers, 49 (70.0%) were from *Salmonella* Dublin–negative farms and 21 (30.0%) were from *Salmonella* Dublin–positive farms. The location of the submitted surveys from the 70 individual farms from the different regions essentially mirrored the location of dairy farms within the province, with the 78.6% of respondents located in the Fraser Valley (n = 55, region contains 69% of BC dairy farms), followed by 11.4% in the North Okanagan (n = 8, 18% of BC dairy farms), 7% within Vancouver Island (n = 5, 8% of BC dairy farms), and 2.9% in Bulkley Valley/Cariboo (n = 2; 4% of BC dairy farms). There were no survey respondents from Creston, which has 2% of BC dairy farms.

In univariable analysis, *Salmonella* Dublin–positive farms had a higher likelihood of feeding pasteurized colostrum to calves *(P* = 0.03) and were less likely to feed raw milk to calves (*P* = 0.04; Table 1). There was no difference (*P* > 0.05) among the remaining risk factors between positive and negative farms.

**Table 1:** Characteristics of *Salmonella* Dublin BTM-negative (n = 49) and BTM-positive farms (n = 21) that completed the study's survey (n = 70 total)

Risk for transmission	Category	Subcategory	Total n (%)	Negative n (%)	Positive n (%)	*P*-value^1^
Risk factors for entering the herd						
	Do cattle ever leave the farm and return?	Yes	19 (27.1)	12 (24.5)	7 (33.3)	0.639
		No	51 (72.8)	37 (75.5)	14 (66.7)	
	House cattle from other farms	Yes	17 (24.3)	12 (24.5)	5 (23.8)	1
		No	53 (75.7)	37 (75.5)	16 (76.2)	
	Open herd (purchase or introduce new cattle)^2^	Yes	45 (77.6)	28 (73.7)	17 (85.0)	0.515
		No	13 (22.4)	10 (26.3)	3 (15.0)	
	Shared pastures	Yes	1 (1.4)	0 (0.0)	1 (4.8)	0.66
		No	69 (98.5)	49 (100.0)	20 (95.2)	
	Regular visitors (veterinarian, artificial insemination tech, hoof trimmer)	Yes	68 (97.1)	48 (98.0)	20 (95.2)	1
		No	2 (2.9)	1 (2.0)	1 (4.8)	
	Enforced protocol for visitors (e.g., change overalls, clean boots)	Yes	36 (51.4)	22 (45.8)	14 (66.7)	0.183
		No	33 (47.1)	26 (54.2)	7 (33.3)	
	Enforced age group order for visitors	Yes	18 (25.7)	15 (31.2)	3 (14.3)	0.239
		No	51 (72.8)	33 (68.8)	18 (85.7)	
	Used heifer rearing	Yes	2 (2.9)	1 (2.1)	1 (4.8)	1
		No	68 (97.1)	47 (97.9)	20 (95.2)	
Risk factors for spread in the herd						
	Newborn removed from mother in <6 h	Yes	52 (74.2)	35 (71.4)	17 (81.0)	0.591
		No	18 (25.7)	14 (28.6)	4 (19.0)	
	More than one adult dairy cow in the maternity pen	Yes	43 (61.4)	26 (53.1)	17 (81.0)	0.054
		No	27 (38.6)	23 (46.9)	4 (19.0)	
	Calves suckle the dam	Yes	49 (70.0)	37 (75.5)	12 (57.1)	0.211
		No	21 (30.0)	12 (24.5)	9 (42.9)	
	Housing sick cattle in calving area	Yes	30 (42.9)	23 (46.9)	7 (33.3)	0.429
		No	40 (57.1)	26 (53.1)	14 (66.7)	
	Feed pasteurized colostrum	Yes	9 (12.8)	3 (6.1)	6 (28.6)	0.029*
		No	61 (87.1)	46 (93.9)	15 (71.4)	
	Feed raw milk to calves	Yes	35 (50.0)	29 (59.2)	6 (28.6)	0.037*
		No	35 (50.0)	20 (40.8)	15 (71.4)	
	House calves in groups vs. pairs/individually	Groups	15 (21.4)	10 (20.4)	5 (23.8)	1
		Pairs/individually	55 (78.5)	39 (79.6)	16 (76.2)	
	Calves can interact with/touch neighbor	Yes	42 (60.0)	31 (63.3)	11 (52.4)	0.558
		No	28 (40.0)	18 (36.7)	10 (47.6)	
	Sick and healthy calves grouped together	Yes	12 (17.1)	7 (14.3)	5 (23.8)	0.533
		No	58 (82.9)	42 (85.7)	16 (76.2)	
	Pens/hutches cleaned between calves	Yes	52 (74.2)	33 (67.3)	19 (90.5)	0.084
		No	18 (25.7)	16 (32.7)	2 (9.5)	
	Equipment shared between calves and adults	Yes	49 (70.0)	37 (75.5)	12 (57.1)	0.211
		No	21 (30.0)	12 (24.5)	9 (42.9)	
	Weaned calves graze with adults^2^	Yes	3 (4.5)	1 (2.1)	2 (9.5)	0.451
		No	64 (97.0)	43 (91.5)	21 (100.0)	
	Standing water access^2^	Yes	4 (5.9)	4 (8.5)	0 (0.0)	0.412
		No	64 (94.1)	43 (91.5)	21 (100.0)	

1 *P*-values were generated through a chi-squared test.

2 Total number of respondents in the category may be <70 due to some producers declining to answer.

* Statistically significant at 5% level of significance.

The variables with a *P*-value <0.20 (Table 1) were considered for the MLR model. Following backward elimination using the AIC, the variables “more than one adult in the maternity pen,” “feeds pasteurized colostrum,” and “cleaning hutches between calves” were selected for inclusion in the final model. There were 2 significant variables within this model. The odds of being *Salmonella* Dublin positive on BTM were 4.28 times higher for farms that housed more than one adult dairy cow in the maternity pen at the same time (odds ratio [**OR**] = 4.28, CI = 1.24–18.4; Table 2). The odds of being *Salmonella* Dublin positive on BTM were 5.01 times higher for farms that pasteurized colostrum (OR = 5.01, CI = 1.048–30.04). The other variable was not significantly different.

**Table 2:** Adjusted odds ratios for variables associated with positive *Salmonella* Dublin status from farms that completed the study's survey (n = 70)

Exposure	Subcategory	Model output	
		OR	95% CI
More than one adult in the maternity pen	No	Referent	
	Yes	4.28	1.24–18.41*
Feed pasteurized colostrum	No	Referent	
	Yes	5.01	1.05–30.54*
Pens/hutches cleaned between calves	No	Referent	
	Yes	3.28	0.71–23.79

* Statistically significant at 5% level of significance.

Overall, the components outlined in the modified Danish tool had limited success differentiating herds that were positive or negative for *Salmonella* Dublin on BTM, with only 2 significant risk factors being identified. Farms that reported housing more than one adult dairy cow in the maternity pen were 4 times more likely to be positive for *Salmonella* Dublin on BTM than farms that only kept one adult in the maternity pen at a time. This is consistent with a similar study conducted in Ontario, which found that farms housing 4 to 6 adult cows in a maternity pen were more 4.5 (CI = 1–21.1) times more likely to be *Salmonella* Dublin–positive than those that housed a maximum of 3 adults in the maternity pen (Perry et al., 2023). Limiting the number of adult cattle in a single maternity pen likely reduces the risk of *Salmonella* Dublin transmission to vulnerable individuals (neonates and calves), thereby reducing the number of infected individuals on the farm. It is well documented that calving is a stressful event that can result in increased shedding of the bacterium; thus, limiting the number of cows housed together at this time should reduce spread (Huzzey et al., 2005; Velasquez-Munoz et al., 2024).

Surprisingly, farms that pasteurized colostrum were more likely to be positive for *Salmonella* Dublin on BTM. This is contrary to multiple other studies, where pasteurization of colostrum has been shown to reduce *Salmonella* Dublin exposure in calves that consume waste milk (Godden et al., 2006; Holschbach and Peek, 2018; Velasquez-Munoz et al., 2024). Because pasteurization has been demonstrated to kill *Salmonella* species from colostrum, it is biologically unlikely for it to be a true risk factor for *Salmonella* Dublin, and therefore the observed association may be due to underlying biases (Godden et al., 2006). For instance, one of the major limitations of this study is the inability to clearly describe the temporality of the implementation of disease management practices (e.g., pasteurizing colostrum) in relation to when the farm became positive on BTM surveillance. Because farms can be infected chronically, the period to which the survey refers may not be the same period in which *Salmonella* Dublin was introduced or spread on the farm (Nielsen and Dohoo, 2013). It is possible that becoming *Salmonella* Dublin positive on BTM is what prompted these farms to introduce disease mitigation practices such as pasteurizing colostrum, which would not be captured in this study given that the farms were already classified as positive or negative before completing the survey. Furthermore, it is possible pasteurization of colostrum is a proxy for another risk factor not captured in this study, such as large herds, which may be more likely to pasteurize colostrum compared with smaller operations (Boyd et al., 2025).

In the Ontario study, farms that had animals leave and return within 2 years were more likely to be *Salmonella* Dublin positive than farms that did not have any animals leave the farm (OR = 2.9, CI = 1.1–7.6; Perry et al., 2023). We were not able to identify this as an association in our study. The question used to evaluate this variable in our study was “Do your cattle ever leave the farm temporarily and return (e.g., to a cattle show, loaned out, vet clinic),” which did not give a timeframe as in the Ontario study (e.g., within 2 years). This may account for the discrepancies, and future surveys should provide a clear time period when asking farmers about their practices. Aside from these discordant findings, all other similar variables (e.g., length of time with dam, feeding calves raw milk) evaluated in both our study and the Ontario study showed no significant differences between positive and negative dairy farms.

The farms completing the survey were proportionally representative of the geographic dairy regions and the proportion of positive and negative farms within BC, which does add strength to the results by providing more generalizable findings and reducing regional biases. Although we only had a 16% response rate, this is similar to other surveys used to evaluate practices on Canadian dairy farms, whose response rates ranged from 7.9% to 12% (Bauman et al., 2016; Denis-Robichaud et al., 2019; Roche et al., 2020). That said, the lower response rate may indicate response biases. Specifically, it is possible that producers that participate in perceived riskier practices (e.g., open herds, heifer rearing) may be less inclined to participate in the survey.

A limitation of this study could be that BTM positivity may be a poor outcome variable to use to capture farm positivity and to test factors that may be associated with *Salmonella* Dublin positivity, as variables may contribute differently to the introduction of *Salmonella* Dublin or on-farm spread. In contrast to the current study, Perry et al. (2023) used a more robust outcome measurement to determine which farms were positive when evaluating the contribution of a similar set of risk factors. Specifically, they required at least one positive serum serology sample from young stock (calves and heifers) as well as a positive BTM sample (Perry et al., 2023). The use of serum serology in young stock may have better accounted for within-farm spread compared with just BTM serology results as young stock do not contribute to the bulk tank and young stock are the most vulnerable to infection (Nielsen, 2013; Boyd et al., 2024). Furthermore, BTM serology has a relatively low herd sensitivity (38%); however, sensitivity can be improved to 95% by repeating 4 tests over 12 mo, which was done during the work to classify our farms (Warnick et al., 2006; Boyd et al., 2024). However, it is possible that the variable sensitivity could have misclassified farms. Given the lack of associations identified in the current study, we suggest that it may be more effective to target newly positive farms detected through BTM surveillance with a similar risk factor survey to better address the issue of temporality, consider a subanalysis that controls for herd size (Boyd et al., 2025), and include an expanded outcome definition of farm positivity that includes sero-status of young stock.

Our attempt to revise and implement the modified Danish risk scoring tool for *Salmonella* Dublin through a survey that included factors specific to Canadian dairy farms exhibited limited success, as only 2 factors that distinguished *Salmonella* Dublin–positive versus *Salmonella* Dublin–negative farms were identified. Future research should seek to determine if further modifications to the survey would be useful, especially the inclusion of questions on factors that target introduction of *Salmonella* Dublin versus on-farm transmission of *Salmonella* Dublin. Alternative outcome measures may also be more effective, such as changes in BTM status, individual cow serology, or clinical *Salmonella* Dublin illness.
